# A Novel Infant Formula with Medium- and Long-Chain Triacylglycerols and *sn*-2 Palmitate Supports Adequate Growth and Lipid Absorption in Healthy Term Infants

**DOI:** 10.3390/nu17091401

**Published:** 2025-04-22

**Authors:** Xiaoyan Chen, Mengtao Yang, Wei Wei, Siyu Huang, Yingzhen Qiu, Zhen Li, Qiuye Lan, Bixia Huang, Tong Wu, Qianqian Bi, Xingguo Wang, Huilian Zhu

**Affiliations:** 1 Guangdong Provincial Key Laboratory of Food, Nutrition and Health, School of Public Health, Sun Yat-sen University, Guangzhou 510080, Chinalanqy@link.cuhk.edu.hk (Q.L.);; 2 State Key Laboratory of Food Science and Resources, Jiangnan University, Wuxi 214122, China

**Keywords:** *sn*-2 palmitate, medium- and long-chain triacylglycerol, adequate growth, lipid absorption, infant formula, human milk, structured lipid

## Abstract

**Background:** Medium- and long-chain triacylglycerols (MLCTs) and *sn*-2 palmitate constitute approximately 70~80% of total breast milk fat. The structured lipid MLCTs and *sn*-2 palmitate, mimicking human milk, have demonstrated improvement in lipid absorption and energy metabolism in vitro and in animal experiments. However, clinical trials on infant formula supplied with MLCTs and *sn*-2 palmitate have yet to be conducted. **Objectives:** This study was designed to evaluate the effects on growth and lipid absorption of a novel formula with structured lipid MLCTs and *sn*-2 palmitate on healthy infants born at term. **Methods:** Infants were enrolled at 30 d postpartum and assigned to three groups based on their feeding conditions: (1) a novel formula with MLCTs and *sn*-2 palmitate (Novel-F group, *n* = 65); (2) a control formula with vegetable oils and no structured lipids (Contr-F group, *n* = 46); or (3) breastfeeding (BF group, *n* = 66). Growth measurements (including weight, length, and head circumference), stool characteristics, and fecal lipid composition (both soap and non-soap fatty acids) were analyzed at both baseline (30 d postpartum) and endline visits (90 d postpartum). **Results:** The Novel-F group had significantly higher weight gains (2195 ± 595 g) during the intervention compared to the Contr-F group (1897 ± 483 g) but similar weight gains to the BF group (2081 ± 614 g), with the changes in Z_w/a_ following a similar pattern. Z_l/a_ increased in the Novel-F group (0.04, (95% CI: −0.21 to 0.28)) and decreased in both the Contr-F (−0.23 (95% CI: −0.52 to 0.06)) and BF groups (−0.20 (95% CI: −0.44 to 0.05)). The stools of infants in the Novel-F group had similar undigested triacylglycerols and total fatty acids compared to breastfed infants but had significantly lower levels than infants fed with the control formula at both baseline and endline visits. **Conclusions:** The novel infant formula with MLCTs and *sn*-2 palmitate is safe and well tolerated, and supports adequate weight gain and improves lipid absorption.

## 1. Introduction

Human milk is the optimal nutritional source for infants, providing adequate calories and nutrients essential for comprehensive growth and development [1]. Human milk lipids, primarily in the form of milk fat globules, serve as a crucial energy source, meeting approximately half of an infant’s energy demands [2]. In human milk, triacylglycerols (TAGs) constitute about 98% of the total fat content and offer abundant medium-chain fatty acids (MCFAs) and long-chain fatty acids (LCFAs) [3]. The distribution of these fatty acids on the glycerol backbone plays a critical role in determining their nutritional value, compatible with infants’ digestion and absorption capacities to support growth and gastrointestinal maturation [4].

TAGs in human milk are widely variable and sophisticated, and they contain various molecular species [5]. Our previous study found that in human milk, MCFAs are mainly esterified with LCFAs, which form medium- and long-chain triacylglycerols (MLCTs), ac-counting for 20% to 40% of the total lipids [6,7]. The MLCTs primarily consist of MCFAs (e.g., C12:0 and C14:0) and LCFAs (e.g., C16:0, C18:1, and C18:2) [8]. In vitro digestion tests have revealed that human milk rich in MLCTs exhibited a higher lipolysis rate after intestinal digestion [7]; infant formula supplied with MLCTs showed moderate MCFA release during gastric digestion and a higher lipolysis degree compared to commercial infant formula without MLCTs [9]. The efficient digestion and absorption of MLCTs meet the high energy demands of infants and are compatible with their immature gastrointestinal systems [10,11]. Additionally, MLCTs have also been found to stimulate fatty acid metabolism in adipose tissue, enhance hepatic β-oxidation, and inhibit visceral fat accumulation, potentially promoting growth and reducing the risk of obesity, as reported by our and other studies [12,13,14,15]. However, infant formula typically consists of a physical mixture of medium-chain TAGs (MCTs) and long-chain TAGs (LCTs) [16], which differs from human milk MLCTs and may lead to different lipid digestion and metabolism [6].

In human milk, *sn*-2 palmitate constitutes approximately 70~75% of the total palmitic acid. Furthermore, *sn*-2 palmitate is a unique type of TAG with palmitic acid esterified at the *sn*-2 position, mainly existing as 1,3-unsaturated fatty acids-2-palmitic acid (UPU)-type TAGs [17]. Fatty acids at the *sn*-1 and *sn*-3 positions are preferentially released after hydrolysis by lipase, while palmitic acid at the *sn*-2 position is less susceptible to hydrolysis, facilitating its absorptions and reducing the formation of calcium soaps [18,19,20,21]. This mechanism minimizes the loss of fatty acids and calcium, thereby supporting bone integrity and the early growth and development of infants [22]. Moreover, *sn*-2 palmitate with oleic acid attached to the *sn*-1/3 positions is known as 1,3-dioleoyl-2-palmitoyl-glycerol (OPO), comprising roughly 15% of the total TAGs found in human milk [8,23]. Furthermore, *sn*-2 palmitate-enriched formula has shown positive effects on growth and gastrointestinal tolerance [24,25,26], including improved stool consistency [27], and the increased comfort and stability of infants [28,29].

In human milk, MLCTs and *sn*-2 palmitate (UPU-type TAGs) together account for the majority of milk lipids (70~80%) and their contents are stable during lactational stages [8]. Our initial research revealed a significant disparity in TAG composition between human milk and infant formula [30]. To address this gap, we have focused on enhancing infant formula to the mimic breast milk lipid composition by incorporating MLCTs and *sn*-2 palmitate. Preliminary in vitro studies have indicated that infant formula supplied with MLCTs and *sn*-2 palmitate exhibits a higher lipid digestion rate [7,9]. Subsequent animal experiments have demonstrated that, compared to commercial formulas containing a physical mixture of MCTs and LCTs, novel milk fat substitutes with MLCTs and *sn*-2 palmitate modulated energy metabolism, the gut microbial composition, and lipid metabolism in mice [16]. These findings suggest that formulas supplied with MLCTs and *sn*-2 palmitate could provide potential benefits for lipid absorption and healthy growth. However, clinical trials are still needed to validate these effects.

Therefore, this study aimed to conduct a prospective, parallel, open-label controlled trial to evaluate the effects of a novel formula supplied with MLCTs and *sn*-2 palmitate on healthy full-term infants compared to regular formula and breastfeeding. We hypothesized that formula fortified with structured lipids MLCTs and *sn*-2 palmitate could support infants’ adequate growth and improve lipid absorption.

## 2. Materials and Methods

### 2.1. Study Design and Subjects

This study was a prospective, parallel, open-label controlled trial conducted from April 2022 to December 2023 in Bozhou, China. Participants were recruited from local communities through posters or researchers’ invitations. During late pregnancy or postpartum, mothers were introduced to the study and encouraged to breastfeed. For those with insufficient breastmilk, infant formula was provided as a supplement. Infants whose mothers confirmed participation within 30 days postpartum and met all criteria were formally enrolled in the study.

Eligible infants met the following criteria: (1) healthy full-term infants (37–42 weeks); (2) single birth; (3) birth weight of between 2500 and 4000 g; (4) Apgar Scores > 7; (5) infants were exclusively breastfed or, if not, were fed one of our study formulas during the fourth week postpartum and consumed more than 80% of the average feeds on days 28, 29, and 30 postpartum; and (6) parental consent was provided with a commitment to adhere to the feeding protocol. Exclusion criteria included the following: (1) infants whose mothers were unable to care for them due to illness or socioeconomic issues; (2) infants with significant congenital anomalies or chromosomal disorders detected at birth; or (3) infants with feeding or metabolic issues due to suspected or unknown metabolic factors. The study was registered at clinicaltrials.gov (NCT05295030) and approved by the Medical Ethics Committee of the School of Public Health, Sun Yat-sen University (no. 2021130). The study was conducted in compliance with principles of the Declaration of Helsinki, with written informed consent was obtained from each mother at enrollment.

Infants were assigned to three groups based on their feeding patterns in the fourth week postpartum. Specifically, those who were fed novel formula containing MLCTs and *sn*-2 palmitate during the fourth week and consuming more than 80% of the average feeds over three days (days 28, 29, and 30 postpartum) were placed into the novel formula group (Novel-F group). Those fed control formula during the fourth week and who were consuming more than 80% of the average feeds over three days (days 28, 29, and 30 postpartum) were placed into the control formula group (Contr-F group). Exclusively breastfed infants were included in the breastfeeding group (BF group).

### 2.2. Trial Diets

Both formulas had similar nutrients. As is shown in Table 1, the novel formula was supplied with structured MLCTs at 4.40 g/100 g and *sn*-2 palmitate at 3.10 g/100 g. These two formulas had similar concentrations of linoleic acid and α-linolenic acid but differed significantly in their total palmitic acid. The ester position profile of palmitic acid (*sn*-2/total PA) in the novel formula was ~40%, which was much higher than in the control formula (~7.2%). Detailed information on the composition of the minerals and trace elements in the two formulas is documented in Table S1.

### 2.3. Intervention Procedures

Infants were enrolled in the Novel-F, Contr-F, or BF groups according to their feeding conditions on day 30 postpartum and were required to continue their respective feeding patterns until day 90 postpartum. At the baseline visit (day 30 postpartum), demographic information, anthropometric assessments, information on stool characteristics, and stool samples were collected at participants’ homes. After the 60-day intervention, these measurements were repeated at the endline visit (day 90 postpartum). Withdrawals, adverse events, and other situations were documented in case report forms.

### 2.4. Outcome Measurements and Sample Size Calculation

The primary outcome comprised the change in anthropometric parameters (weight, length, and head circumference) from the baseline to the end of the study. Secondary outcomes were constituted by changes in stool characteristics (frequency and consistency) and fecal lipid composition. The sample size calculation referenced data from the American Academy of Pediatrics (AAP), detecting a 3 g/day weight gain difference (SD = 4.5 g/day). Therefore, each group required 37 infants (α  =  0.05, power  =  0.81). Accounting for a 20% expected dropout, 47 infants per arm were planned for enrollment.

### 2.5. Collection of Participant Information and Stools

Demographic information for infants and mothers, including gestational age, gender, delivery mode, birth weight, birth length, maternal age, primipara status, and tobacco exposure during pregnancy, was collected from the obstetric medical records and questionnaires at enrollment. At both the baseline and endline visits, infants’ length, weight, and head circumference were measured by qualified pediatricians. Weight-for-age *z*-scores (Z_w/a_), length-for-age *z*-scores (Z_l/a_), weight-for-length *z*-scores (Z_w/l_), and head circumference-for-age *z*-scores (Z_c/a_) were calculated according to the Chinese growth standards [31]. The changes in anthropometry from baseline were calculated using the following equation: change = endline − baseline [32]. Three-day diaries were used to record daily formula intake, stool characteristics (frequency and consistency), gastrointestinal intolerance (diarrhea, difficulty in defecating, spitting up, and postprandial crying), and adverse events at both the baseline and endline visits. The stool consistency score was evaluated using the simplified Bristol Stool Form Scale [33], in which parents ranked each stool from type 1 to type 6 (type 1: separate hard lumps; type 2: sausage-shaped but lumpy; type 3: formed but with cracks; type 4: smooth and soft; type 5: soft blobs with clear-cut edges; type 6: mushy). Stool samples collections were performed at both the baseline and endline visits. The samples were collected at home using sterile tubes, which were stored at −20 °C and promptly transported to the lab. The samples were then frozen at −80 °C for subsequent fecal lipid analysis. All data were collected by uniformly trained researchers following standardized operating procedures. Questionnaire data were obtained through in-person interviews conducted by our team. For stool consistency score assessments and sample collection, parents received comprehensive face-to-face training, detailed guidance, and an operational manual to ensure consistency and accuracy.

### 2.6. The Non-Soap Fatty Acids and Triacylglycerol Analysis

The non-soap fatty acids, soap fatty acids, and TAGs were extracted and separated using the Soxhlet extraction method, as described previously [34,35], with modifications. Briefly, approximately 1 g of the stool sample was frozen in a −40 °C refrigerator and then freeze-dried for 24 h to obtain dry matter. The accurate weighed frozen dry sample (approximately 0.2 g) was ground to a powder, and then the lipids were extracted by the FOSS automatic Soxhlet extraction system (FOSS Scino Co., Ltd., Hilloeroed, Denmark) with 40 mL of petroleum ether (30 °C to 60 °C) at 80 °C, with a reflux time of 3.5 h. Then, the petroleum ether phase was transferred. After removing the organic solvents to achieve a constant weight, the samples were then weighted and maintained at −20 °C pending subsequent analysis.

The non-soap fatty acids and TAGs were then analyzed using the Waters 1525 HPLC-2414 RID (Waters, Milford, MA, USA)system as previously described [36]. The column used was a Sepax HP-Silica column (4.6 mm × 250 mm × 5 μm, Sepax Technologies, Newark, DE, USA). The mobile phase consisted of a mixture of n-hexane/isopropyl alcohol/formic acid (15:1:0.003, by volume). Lipid classes including TAGs, diacylglycerols, monoacylglycerol, and free non-soap fatty acids were identified according to the retention time previously described [36] and quantified in terms of concentration in dry matter (% dry matter).

### 2.7. The Soap Fatty Acid Analysis

After the Soxhlet extraction described above, the residue was transferred to 50 mL centrifuge tubes with 5 mL of 3 mol/L HCl solution and 10 mL petroleum ether added. After being sufficiently mixed, the mixture was ultrasonicated (20 min), followed by centrifugation (4000 rpm, 5 min). The organic supernatant was transferred, and 10 mL of petroleum ether was added again to repeat the above operation. The organic phases were combined and the organic solvent was removed to obtain the soap fatty acid samples. The samples were subsequently weighed and held at −20 °C until analysis.

The soap fatty acid samples were analyzed using UPLC-Q-TOF-MS following the method outlined in our previous publication [9]. Briefly, lipid separation was performed using a UPLC BEH C18 (2.1 mm × 150 mm × 1.7 μm) column maintained at 65 °C. The mobile phases comprised (A) acetonitrile/iso-propanol (1:9, *v*/*v*) and (B) acetonitrile/water (4:6, *v*/*v*), both containing 10 mmol/L of ammonium acetate. The Q-TOF-MS was operated in the negative ion electrospray ionization (ESI) mode, and the scan range was 100~1500 *m*/*z*. Acquired data were analyzed using Waters MassLynx v4.1.

### 2.8. Statistical Analysis

All analyses were performed according to the intention-to-treat (ITT) methodology. For demographic characteristics, one-way ANOVA was conducted to compare continuous variables, while chi-square tests were employed for categorical variables. Additionally, two-way repeated-measures ANOVA was performed to compare differences in primary-outcome variables, including growth measurements and their *z*-scores. One-way ANOVA was applied to assess differences in secondary-outcome variables, including stool characteristics (frequency and consistency) and fecal lipid content (non-soap fatty acids, soap fatty acids, total fatty acids, and TAGs). The Bonferroni test was used for multiple comparisons. Stool consistency types were analyzed by Fisher’s exact test. Statistical computations were implemented using IBM SPSS 27.0. All tests were two-sided, with α = 0.05.

## 3. Results

### 3.1. Baseline Characteristics

**Figure 1:**
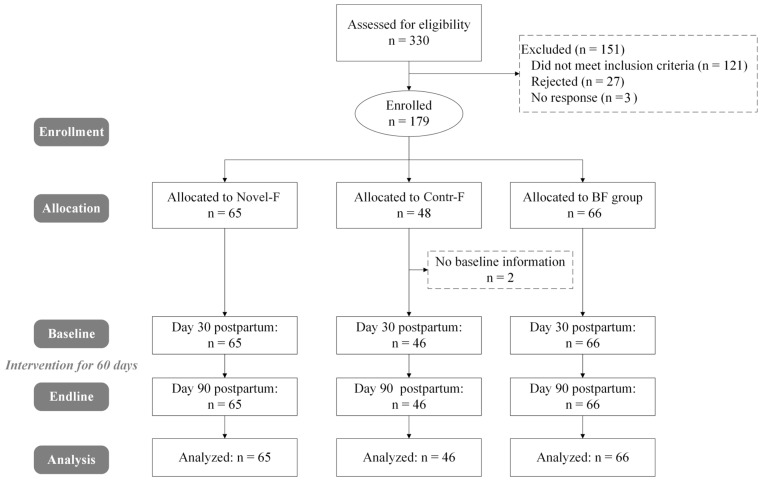
Flowchart of this study.

As is shown in Figure 1, a total of 330 participants were assessed for eligibility, among whom 151 were excluded (121 did not meet the inclusion criteria, 27 declined, and 3 did not respond). A total of 179 healthy full-term infants were allocated to the interventions (Novel-F group, *n* = 65; Contr-F group, *n* = 48; BF group, *n* = 66). However, baseline information for two infants in the Contr-F group was not obtained and they were excluded from further analysis. During the follow-up period, no participant withdrew from the trial. Finally, 177 participants completed the endline visit with completed data, including 65 in the Novel-F group, 46 in the Contr-F group, and 66 in the BF group.

The baseline characteristics of participants were similar between the Novel-F and Contr-F groups. Infants in the Novel-F group had a lower gestational age compared with the BF group. Factors such as the gender of newborns, birth weight, birth length, maternal age, delivery mode, primipara status, and tobacco exposure during pregnancy remained comparable between the Novel-F and BF groups (Table 2).

### 3.2. Effect of Novel Formula Supplied with MLCTs and sn-2 Palmitate on Growth

The growth measurements of infants are presented in Table 3. No significant differences were observed in these measurements between the groups at baseline. After the intervention, infant weights in the Novel-F, Contr-F, and BF groups were 6705 ± 580 g, 6362 ± 561 g, and 6645 ± 723 g, respectively. Infants in the Novel-F group demonstrated a significantly higher weight compared to the Contr-F group, with a comparable weight to the BF group. The weight changes were significantly higher in the Novel-F group (2178 g (95% CI: 2038 g to 2319 g)) compared to the Contr-F group (1885 g (95% CI: 1718 g to 2052 g)), with no significant difference compared to the BF group (2100 g (95% CI: 1960 g to 2241 g)). Additionally, the Novel-F group demonstrated a comparable length and head circumference to both the Contr-F and BF groups.

The *z*-scores for the anthropometric measures in all groups aligned with the growth curves, as shown in Table 4. After the intervention, Z_w/a_ in the Novel-F group (0.26 ± 0.77) was significantly higher than the Contr-F group (−0.26 ± 0.76). Additionally, Z_l/a_ increased in the Novel-F group (0.04 (95% CI: −0.21 to 0.28)) and decreased in both the Contr-F (−0.23 (95% CI: −0.52 to 0.06)) and BF groups (−0.20 (95% CI: −0.44 to 0.05)), although no significant difference was observed between the groups. Specifically, Z_l/a_ increased in 55.4%, 34.8% and 43.9% of infants in the Novel-F, Contr-F, and BF group, respectively (Figure S1). Furthermore, the increase in Z_w/l_ in the Novel-F group (0.47 (95% CI: 0.12 to 0.82)) was closer to the BF group (0.46 (95% CI: 0.11 to 0.80)) and slightly higher than that of the Contr-F group (0.17 (95% CI: −0.25 to 0.58)), though this was not statistically significant.

### 3.3. Stool Characteristics

In this study, stool characteristics, including frequency and consistency, were collected at both baseline and endline visits (Table 5). Stool frequency and consistency scores in the Novel-F group were similar to the Contr-F group but lower than those in the BF group at both time points. Similar results were observed for the stool consistency types (Table 6), with type 6 stools being predominant at baseline (Novel-F group, 61.5%; Contr-F group 73.9%; and BF group, 80.3%), followed by type 5 stools (Novel-F group, 21.5%; Contr-F group, 19.6%; and BF group, 19.7%). At the end of study, the proportion of type 6 stools decreased (Novel-F group, 40.0%; Contr-F group, 32.6%; and BF group, 68.2%) and type 5 stools increased (Novel-F group, 50.8%; Contr-F group, 60.9%; and BF group, 28.8%). Notably, there were two infants for whom type 1 and type 2 stools disappeared after the intervention in the Novel-F group, indicating an improvement in stool dryness. Symptoms of gastrointestinal intolerance were observed in a few cases at baseline, including diarrhea, difficulty in defecating, spitting up, and postprandial crying (Table S2). No cases of gastrointestinal infectious diseases were reported in any group (Table S2).

### 3.4. Fecal Lipid Composition

The TAG contents at baseline of the Novel-F group, Contr-F group, and BF group were 5.36 ± 3.92%, 10.01 ± 8.32%, and 6.04 ± 2.90%, respectively (Table S3 and Figure S2). The TAG content did not differ significantly between the Novel-F and BF groups (*p* = 0.305); in contrast, the content in the Novel-F group was significantly lower compared to the Contr-F group (*p* < 0.001). After the intervention at 90 d postpartum, the TAG content generally decreased compared to 30 d postpartum. The TAG content in the Novel-F group was significantly lower than in the Contr-F group (4.21 ± 2.55% vs. 7.14 ± 7.41%, *p* = 0.005) and similar to that in the BF group (5.08 ± 3.45%, *p* = 0.140).

In this study, both non-soap and soap fatty acids were analyzed and they are shown in Table S3 and Table 7. At 30 d postpartum, the non-soap-fatty-acid contents of the Novel-F group, Contr-F group, and BF group were 16.31 ± 9.62%, 20.93 ± 10.50%, and 18.30 ± 7.91%, respectively, with no significant differences observed among the three groups. At 90 d postpartum, the fecal non-soap-fatty-acid contents in the Novel-F, Contr-F, and BF groups were 12.82 ± 6.66%, 17.73 ± 8.99%, and 17.87 ± 11.61%, respectively, with the content in the Novel-F group being lower than that in the Contr-F group (*p* = 0.008) and BF group (*p* = 0.017). At the baseline visit, the soap-fatty-acid contents of the Novel-F and Contr-F groups were both significantly higher compared with the BF group. However, at the endline visit, no differences were observed in the soap-fatty-acid content among the three groups. In addition, Table 7, Table S3, and Figure S2 also show the total fatty acids in infant stools, which represent the sum of soap and non-soap fatty acids. At both the baseline and endline visits, the total fatty acid content was significantly lower in the Novel-F group than the Contr-F group (baseline: 33.18 ± 13.36% vs. 39.39 ± 16.94%, *p* = 0.031; endline: 30.46 ± 10.22% vs. 35.68 ± 14.87%, *p* = 0.024), with no differences observed between the Novel-F and BF groups.

The molecular species of soap fatty acids were further analyzed using LC–MS (Table S3 and Table 7), identifying five saturated fatty acids and five unsaturated fatty acids. Soap 16:0, and soap 18:0 were major soap fatty acids and were significantly higher in both the Novel-F and Contr-F groups compared to the BF group. The main unsaturated soap fatty acids were 18:1 and 18:2. For soap 18:1, the content in the Novel-F group was closer to that in the BF group and lower than in the Contr-F group; however, no significant differences were observed. Additionally, we detected soap long-chain polyunsaturated fatty acids (e.g., DHA) in infant stools, which were present in low amounts and showed larger individual differences.

## 4. Discussion

This study was the first to evaluate the effects of fortified formula supplemented with MLCTs and *sn*-2 palmitate on healthy full-term infants regarding growth and lipid absorption. These two types of structured lipids together account for the majority of total TAGs in human milk. Our results demonstrated that infants in the Novel-F group had higher weight gains compared to the Contr-F group, whereas similar weight gains were observed compared with the BF group. The change in Z_w/a_ also followed this pattern, indicating that MLCT- and *sn*-2 palmitate-enriched formula supports adequate weight gain. Additionally, a higher proportion of infants in the Novel-F group showed an increase in Z_l/a_, suggesting that this formula may also have potential benefits for growth in terms of length. Furthermore, lower concentrations of undigested TAGs and total fatty acids were observed in the Novel-F group relative to the Contr-F group, suggesting better lipid absorption for infants fed with novel formula enriched with MLCTs and *sn*-2 palmitate.

Consistent with previous studies [37,38,39], a higher weight gain was reported with the feeding of fortified formula containing *sn*-2 palmitate, although no statistically significant difference was observed versus the control group in those studies. The pronounced weight gain in our study may be due to the synergy of MLCTs and *sn*-2 palmitate. These two structured lipid components account for the majority of total TAGs in human milk [8], providing sufficient calories to sustain normal growth [8]. The *sn*-2 palmitate is known to enhance fat absorption, reduce fat oxidation, and lower daily energy expenditure, resulting in weight gain [40,41]. On the other hand, the structured lipid MLCTs, in contrast to a physical mixture of MCTs and LCTs, may promote energy expenditure, regulate lipid metabolism, and inhibit the de novo synthesis of fatty acids, helping to prevent excessive weight gain [42,43,44]. Therefore, MLCT- and *sn*-2 palmitate-fortified formula supports adequate weigh gain comparable to that of breastfed infants. The similar changes in Z_l/a_ and Z_w/l_ observed between the Novel-F and BF groups suggest potential benefits for increased length as well. Furthermore, TAGs and total fatty acids in infant stools reflect the undigested part of the ingested lipids. The stools of infants fed the novel formula with MLCTs and *sn*-2 palmitate showed similar undigested lipids to those among breastfed infants, but lower levels to those fed the control formula. These findings indicate that formula containing MLCTs and *sn*-2 palmitate may promote adequate weight gain in infants by improving lipid absorption [45].

Our results also demonstrated that the formula enriched with MLCTs and *sn*-2 palmitate improved lipid absorption and was well tolerated, consistent with previous studies [27,46,47]. Undigested fatty acids in infant stools exist in both non-soap and soap forms. Soap fatty acids are generally saturated fatty acids that bind with calcium and magnesium ions to form insoluble soap under the conditions of the infant gastrointestinal tract [20,48]. *Sn*-2 palmitate can reduce calcium soap formation [27], as palmitic acid in *sn*-2 palmitate is less prone to hydrolyze from the glycerol skeleton [20]. Additionally, the calcium soaps found in stools primarily consist of LCFA calcium soaps, and their formation depends on the content of LCFAs [49]. Compared to LCTs, MLCTs contain a lower proportion of LCFAs, which may reduce the formation of calcium soap. This facilitates the absorption of fatty acids and calcium in the intestine, reduces insoluble calcium soaps [20], and thereby supports growth and improves gastrointestinal comfort. In this study, soap 16:0 and soap 18:0 were the major soap fatty acids showing no differences observed between infant stools in the Novel-F and Contr-F groups, possibly due to the significantly higher palmitic acid content in the novel formula than the control formula (Table 1). Additionally, despite the higher concentration of palmitic acid in the novel formula, it did not exhibit a higher level of soap fatty acids, but showed lower levels of non-soap fatty acids compared to the Contr-F group. This indicates that the higher proportion of *sn*-2 palmitic in the novel formula may facilitate the efficient absorption of saturated fatty acids. These findings further suggest that the combination of *sn*-2 palmitate and MLCTs supports infant growth and gastrointestinal tolerance by enhancing lipid digestion and absorption.

This study has several limitations. Firstly, we did not implement randomization and blindness due to ethical reasons. We rigorously established and adhered to the inclusion and exclusion criteria, while recruiting as many subjects as possible and not just meeting the minimum required sample size. On the other hand, anthropometric measurements and fecal lipid detection were conducted by trained investigators who were unaware of the group assignments, ensuring the reliability of the results. Secondly, the short intervention period limited our ability to assess the sustained effects of MLCT- and *sn*-2 palmitate-supplemented formula on growth and lipid absorption. Additional long-term investigations are needed to comprehensively understand the potential benefits associated with formula containing MLCTs and *sn*-2 palmitate. Thirdly, stool characteristics were reported by parents. Although stool charts were used to guide them, this approach may still introduce reporting bias. Future studies should encourage parents to provide photos for researchers to assess the stool characteristics directly. Additionally, blood samples were not collected in this study due to practical challenges and ethical consideration in newborns. The short-chain fatty acids were not detected using LC–MS, which also need to discussed in a future study. Lastly, only the molecular species of soap fatty acids were studied. Lipidomic analysis of stool samples may add further insights to elucidate the beneficial effects observed in this study.

## 5. Conclusions

In conclusion, this study indicated that formula enriched with MLCTs and *sn*-2 palmitate was well tolerated, supported adequate weight gain, and may offer potential benefits in terms of increased length by enhancing lipid absorption. These findings suggest that formula supplemented with MLCTs and *sn*-2 palmitate is a viable and practical alter-native for infants who cannot be breastfed. Further research should explore its long-term effects on infant health.

## Figures and Tables

**Table 1:** Macronutrients and lipid profiles of the study formulas (per 100 g).

	Novel-F	Contr-F
Energy, kJ	2140	2119
Carbohydrate, g	52.78	52.40
Protein, g	11.52	10.78
Fat, g	27.48	27.50
*sn*-2 palmitate, g	3.10	/
MLCTs, g	4.40	/
Linoleic acid, g	4.79	4.52
α-linolenic acid, mg	404	452
Oleic acid, g	8.38	11.3
Palmitic acid, g	7.18	1.73
*sn*-2 palmitic acid/total palmitic acid, %	41.5	7.2

Abbreviations: MLCTs, medium- and long-chain triacylglycerols.

**Table 2:** Baseline characteristics of participants.

Characteristics	Novel-F (*n* = 65)	Contr-F (*n* = 46)	BF (*n* = 66)
Gestational age, weeks	39.1 ± 1.3 ^a^	39.4 ± 1.4 ^a^	40.3 ± 1.4 ^b^
Male infants, *n* (%)	36 (55.4)	27 (58.7)	38 (57.6)
Birth weight, g	3291 ± 390	3294 ± 388	3429 ± 336
Birth length, cm	50.21 ± 1.28	50.35 ± 1.20	50.54 ± 1.64
Maternal age, years	28.92 ± 4.91	28.59 ± 4.85	27.61 ± 4.40
Vaginal delivery, *n* (%)	26 (40.0) ^ab^	28 (60.9) ^b^	25 (37.9) ^a^
Primipara, *n* (%)	18 (27.7)	16 (34.8)	28 (42.4)
Tobacco exposure during pregnancy, *n* (%)	20 (30.8)	12 (26.1)	25 (37.9)
Infant DHA intake, mg/d	131.6 ± 30.6	128.9 ± 26.3	125.0 ± 35.4

Mean ± SD for continuous variables and *n* (%) for categorical variables. One-way ANOVA was used to compare continuous variables and chi-square tests were used for categorical variables. Different superscript letters (a–b) indicate significant differences (*p* < 0.005 with Bonferroni test).

**Table 3:** Weight, length, and head circumference of infants during the intervention.

	Novel-F (*n* = 65)	Contr-F (*n* = 46)	BF (*n* = 66)	*p*		
				Group	Time	Interaction
Weight, g						
Baseline, mean (SD)	4510 ± 490	4465 ± 492	4565 ± 444	0.052	<0.001	0.028
Endline, mean (SD)	6705 ± 580 ^a^	6362 ± 561 ^b^	6645 ± 723 ^ab^			
Change, mean (95% CI)	2178 (2038 to 2319)	1885 (1718 to 2052)	2100 (1960 to 2241)			
Length, cm						
Baseline, mean (SD)	55.1 ± 2.0	55.0 ± 1.9	55.0 ± 1.6	0.171	0.004	0.274
Endline, mean (SD)	62.2 ± 2.3	61.4 ± 2.0	61.5 ± 2.3			
Change, mean (95% CI)	7.1(6.5 to 7.6)	6.5 (5.8 to 7.1)	6.5 (6.0 to 7.0)			
Head circumference, cm						
Baseline, mean (SD)	36.8 ± 0.9	36.5 ± 0.8	36.8 ± 1.1	0.132	<0.001	0.11
Endline, mean (SD)	40.3 ± 0.9	40.1 ± 0.7	40.0 ± 0.9			
Change, mean (95% CI)	3.5(3.2 to 3.7)	3.5 (3.3 to 3.8)	3.2 (3.0 to 3.4)			

Two-way repeated-measures ANOVA was performed to compare differences among groups. Different superscript letters (a–b) indicate significant differences (*p* < 0.05 with Bonferroni test).

**Table 4:** *Z*-scores of weight, length, and head circumference of infants during the intervention.

	Novel-F (*n* = 65)	Contr-F (*n* = 46)	BF (*n* = 66)	*p*		
				Group	Time	Interaction
Z_w/a_						
Baseline, mean (SD)	0.09 ± 1.00	−0.02 ± 0.92	0.18 ± 0.89	0.047	0.142	0.042
Endline, mean (SD)	0.26 ± 0.77 ^a^	−0.26 ± 0.76 ^b^	0.14 ± 0.90 ^ab^			
Change, mean (95% CI)	0.13 (−0.08 to 0.35)	−0.28 (−0.54 to −0.03)	0.02 (−0.19 to 0.23)			
Z_l/a_						
Baseline, mean (SD)	0.21 ± 0.97	0.14 ± 0.85	0.17 ± 0.75	0.145	0.016	0.275
Endline, mean (SD)	0.26 ± 0.97	−0.09 ± 0.92	−0.05 ± 1.00			
Change, mean (95% CI)	0.04 (−0.21 to 0.28)	−0.23 (−0.52 to 0.06)	−0.20 (−0.44 to 0.05)			
Z_w/l_						
Baseline, mean (SD)	−0.33 ± 0.85	−0.29 ± 1.25	−0.08 ± 1.00	0.058	0.001	0.478
Endline, mean (SD)	0.14 ± 0.92	−0.92 ± 0.98	0.37 ± 1.15			
Change, mean (95% CI)	0.47 (0.12 to 0.82)	0.17 (−0.25 to 0.58)	0.46 (0.11 to 0.80)			
Z_c/a_						
Baseline, mean (SD)	0.13 ± 0.76	−0.15 ± 0.62	0.08 ± 0.86	0.055	0.746	0.103
Endline, mean (SD)	0.22 ± 0.74	−0.03 ± 0.47	−0.08 ± 0.71			
Change, mean (95% CI)	0.10 (−0.10 to 0.29)	0.12 (−0.11 to 0.35)	−0.16 (−0.35 to 0.35)			

Data are presented as mean ± SD; differences among groups were analyzed by two-way repeated-measures ANOVA, with significant differences (*p* < 0.05 with Bonferroni test) denoted by distinct superscript letters (a–b). Abbreviations: Z_w/a_, weight-for-age *z*-scores; Z_l/a_, length-for-age *z*-scores; Z_w/l_, weight-for-length *z*-scores; Z_c/a_, head circumference-for-age *z*-scores.

**Table 5:** Stool characteristics of infants under each feeding pattern.

	Novel-F (*n* = 65)	Contr-F (*n* = 46)	BF (*n* = 66)
Frequency			
Baseline	1.40 ± 0.86 ^a^	1.50 ± 0.83 ^ab^	1.99 ± 1.66 ^b^
Endline	0.94 ± 0.47 ^a^	1.10 ± 0.44 ^ab^	1.28 ± 0.88 ^b^
Change	−0.45 ± 0.98	−0.40 ± 0.94	−0.71 ± 1.58
Consistency			
Baseline	5.34 ± 1.06 ^a^	5.65 ± 0.67 ^ab^	5.80 ± 0.40 ^b^
Endline	5.31 ± 0.64 ^a^	5.24 ± 0.64 ^a^	5.65 ± 0.54 ^b^
Change	−0.03 ± 1.13	−0.41 ± 0.88	−0.15 ± 0.64

Data are presented as mean ± SD; differences among groups were analyzed by one-way ANOVA, with significant differences (*p* < 0.05 with Bonferroni test) denoted by distinct superscript letters (a–b). Change = Endline − Baseline.

**Table 6:** Percentage of stool consistency types among infants in each feeding pattern.

	Novel-F (*n* = 65)	Contr-F (*n* = 46)	BF (*n* = 66)	*p*
Baseline				0.055
Type 1	1 (1.5)	0 (0.0)	0 (0.0)	
Type 2	1 (1.5)	0 (0.0)	0 (0.0)	
Type 3	2 (3.1)	1 (2.2)	0 (0.0)	
Type 4	7 (10.8)	2 (4.3)	0 (0.0)	
Type 5	14 (21.5)	9 (19.6)	13 (19.7)	
Type 6	40 (61.5)	34 (73.9)	53 (80.3)	
Endline				<0.001
Type 1	0 (0.0)	0 (0.0)	0 (0.0)	
Type 2	0 (0.0)	0 (0.0)	0 (0.0)	
Type 3	0 (0.0)	1 (2.2)	0 (0.0)	
Type 4	6 (9.2)	2 (4.3)	2 (3.0)	
Type 5	33 (50.8)	28 (60.9)	19 (28.8)	
Type 6	26 (40.0)	15 (32.6)	45 (68.2)	

Stool consistency types were categorized as follows: type 1, separate hard lumps; type 2, sausage-shaped but lumpy; type 3, formed but with cracks; type 4, smooth and soft; type 5, soft blobs with clear-cut edges; type 6, mushy; data are presented as *n* (%); differences between groups were analyzed by Fisher’s exact test.

**Table 7:** The content of non-soap fatty acids, triacylglycerols, and soap fatty acids in the stools (% dry matter) of infants at endline visit.

Lipids	Novel-F	Contr-F	BF
Non-soap fatty acids	12.82 ± 6.66 ^a^	17.73 ± 8.99 ^b^	17.87 ± 11.61 ^b^
Soap fatty acids	16.07 ± 7.57 ^b^	17.17 ± 8.33 ^b^	12.13 ± 9.10 ^a^
12:0	0.01 ± 0.03	0.02 ± 0.04	0.01 ± 0.03
14:0	0.18 ± 0.12 ^ab^	0.21 ± 0.27 ^b^	0.14 ± 0.21 ^a^
16:0	8.70 ± 4.82 ^b^	7.86 ± 6.57 ^b^	4.99 ± 4.33 ^a^
18:2	0.28 ± 0.24	0.28 ± 0.25	0.29 ± 0.36
18:1	1.75 ± 1.17 ^ab^	1.87 ± 1.55 ^b^	1.33 ± 1.27 ^a^
18:0	4.28 ± 1.51 ^ab^	4.57 ± 2.62 ^b^	3.59 ± 2.20 ^a^
20:4	0.07 ± 0.09	0.05 ± 0.05	0.11 ± 0.22
22:6	0.06 ± 0.09	0.03 ± 0.05	0.30 ± 1.72
24:0	0.78 ± 0.59 ^ab^	0.89 ± 0.85 ^b^	0.64 ± 0.96 ^a^
24:1	0.05 ± 0.06	0.07 ± 0.05	0.08 ± 0.11
Total fatty acids	30.46 ± 10.22 ^a^	35.68 ± 14.87 ^b^	30.06 ± 15.59 ^ab^
Triacylglycerols	4.21 ± 2.55 ^a^	7.14 ± 7.41 ^b^	5.08 ± 3.45 ^ab^

Data are presented as mean ± SD; differences among groups were analyzed using one-way ANOVA, with significant differences (*p* < 0.05 with Bonferroni test) denoted by distinct superscript letters (a–b).

## Data Availability

Data described in the manuscript will not be made available because approval has not been granted by the study participants.

## Supplementary Materials

The following supporting information can be downloaded at: https://www.mdpi.com/article/10.3390/nu17091401/s1, Table S1: Detailed information on composition of the study formulas; Table S2: Gastrointestinal tolerance of infants in each feeding pattern; Table S3: The content of nonsoap fatty acids, triacylglycerol, and soap fatty acids in the stools (% dry matter) of infants at baseline; Figure S1: *Z*-score changes on growth measurements from baseline (30 d postpartum) to the end of the study (90 d postpartum). (A) weight-for-age *z*-scores (Z_w/a_); (B) length-for-age *z*-scores (Z_l/a_); (C) weight-for-length *z*-scores (Z_w/l_); (D) head circumference-for-age *z*-scores (Z_c/a_); Figure S2: (A) Total fatty acids and (B) triacylglycerols (% of dry matter) infant stools measurements at 30 d postpartum and 90 d postpartum. Values were expressed as mean ± SD. One-way ANOVA was used to compare differences between groups and Bonferroni test was applied to compare the Novel-F group and the Contr-F or BF groups, respectively; * and *** indicated *p* < 0.05 and *p* < 0.001, respectively.

## Abbreviations

MLCTs	Medium- and long-chain triacylglycerols
TAGs	Triacylglycerols
MCFAs	Medium-chain fatty acids
LCFAs	Long-chain fatty acids
LCTs	Long-chain TAGs
OPO	1,3-dioleoyl-2-palmitoyl-glycerol
UPU	1,3-unsaturated fatty acids-2-palmitic acid
Novel-F	Novel formula group
Contr-F	Control formula group
BF group	Breastfeeding group
Z_w/a_	Weight-for-age *z*-scores
Z_l/a_	Length-for-age *z*-scores
Z_w/l_	Weight-for-length *z*-scores
Z_c/a_	Head circumference-for-age *z*-scores
HPLC	High-performance liquid chromatography
RID	2414 Refractive Index Detector
ITT	Intention-to-treat
