# Supplementary material for: A Novel Infant Formula with Medium- and Long-Chain Triacylglycerols and sn-2 Palmitate Supports Adequate Growth and Lipid Absorption in Healthy Term Infants
Source: Nutrients. 2025 Apr 22;17(9):1401. doi: 10.3390/nu17091401 (PMC12074145; doi:10.3390/nu17091401)
Supplement: Supplementary file 1 [file nutrients-17-01401-s001.zip › nutrients-3560501-supplementary.pdf]

Table S1. Detailed information on composition of the study formulas.

	Novel-F (per 100g)	Contr-F (per 100g)
GOS, g	3.38	2.95
FOS, g	0.38	0.32
Vitamins		
Vitamin A, µgRE	424	464
Vitamin D, µg	11.80	7.66
Vitamin E, mg α-TE	9.35	8.54
Vitamin K_1, µg	56.1	58.9
Vitamin B_1, µg	581	722
Vitamin B_2, µg	1232	1208
Vitamin B_6, µg	329	385
Vitamin B_{12}, µg	2.10	1.54
Vitamin C, mg	59.0	79.2
Minerals		
Sodium, mg	211	159
Kalium, mg	437	475
Copper, µg	399.0	353.4
Magnesium, mg	47.5	37.1
Iron, mg	5.51	5.23
Zinc, mg	3.90	4.18
Manganese, µg	57.0	88.4
Calcium, mg	304	391
Phosphorus, mg	203	260
Iodine, µg	85.5	81.0
Chlorine, mg	334	380
Selenium, µg	22.7	16.2

Abbreviations: GOS, galacto-oligosaccharides; FOS, fructo-oligosaccharides.

Table S2. Gastrointestinal tolerance of infants in each feeding pattern.

	Novel-F<br>(*n* = 65)	Contr-F<br>(*n* = 46)	BF<br>(*n* = 66)
**Diarrhea, *n* (%)**			
Baseline	3 (4.6)	1 (2.2)	1 (1.5)
Endline	0 (0.0)	0 (0.0)	1 (1.5)
**Difficulty in defecation, *n* (%)**			
Baseline	0 (0.0)	2 (4.3)	0 (0.0)
Endline	1 (1.5)	0 (0.0)	2 (3.0)
**Spitting up, *n* (%)**			
Baseline	0 (0.0)	1 (2.2)	0 (0.0)
Endline	0 (0.0)	0 (0.0)	0 (0.0)
**Postprandial crying, *n* (%)**			
Baseline	0 (0.0)	0 (0.0)	1 (1.5)
Endline	0 (0.0)	0 (0.0)	1(1.5)

Table S3. The content of nonsoap fatty acids, triacylglycerol, and soap fatty acids in the stools (% dry matter) of infants at baseline.

Lipids	Novel-F	Contr-F	BF
Nonsoap fatty acids	16.31±9.62	20.93±10.50	18.30±7.91
Soap fatty acids	16.15±9.55^{b}	17.70±10.24^{b}	9.83±6.46^{a}
12:0	0.02±0.03^{a}	0.03±0.05^{b}	0.01±0.02^{a}
14:0	0.17±0.14^{ab}	0.23±0.21^{b}	0.13±0.15^{a}
16:0	8.30±5.17^{b}	8.15±5.78^{b}	4.34±3.32^{a}
18:2	0.28±0.22^{a}	0.41±0.36^{b}	0.37±0.33^{ab}
18:1	1.85±1.52^{ab}	2.44±1.66^{b}	1.76±1.69^{a}
18:0	4.45±2.02^{b}	4.90±3.12^{b}	3.33±1.76^{a}
20:4	0.06±0.05^{a}	0.08±0.09^{ab}	0.09±0.06^{b}
22:6	0.05±0.07	0.05±0.08	0.04±0.04
24:0	0.90±0.83^{b}	1.15±0.95^{b}	0.50±0.71^{a}
24:1	0.10±0.25	0.13±0.14	0.10±0.10
Total fatty acids	33.18±13.36^{a}	39.39±16.94^{b}	29.91±11.97^{ab}
Triacylglycerols	5.36±3.92^{a}	10.01±8.32^{b}	6.04±2.90^{ab}

The values were expressed as mean ± SD; One-way ANOVA was used to compare differences between groups. Different superscript letters (a–b) indicate significant differences of the values presented in the row ($p < 0.05$ with Bonferroni test).

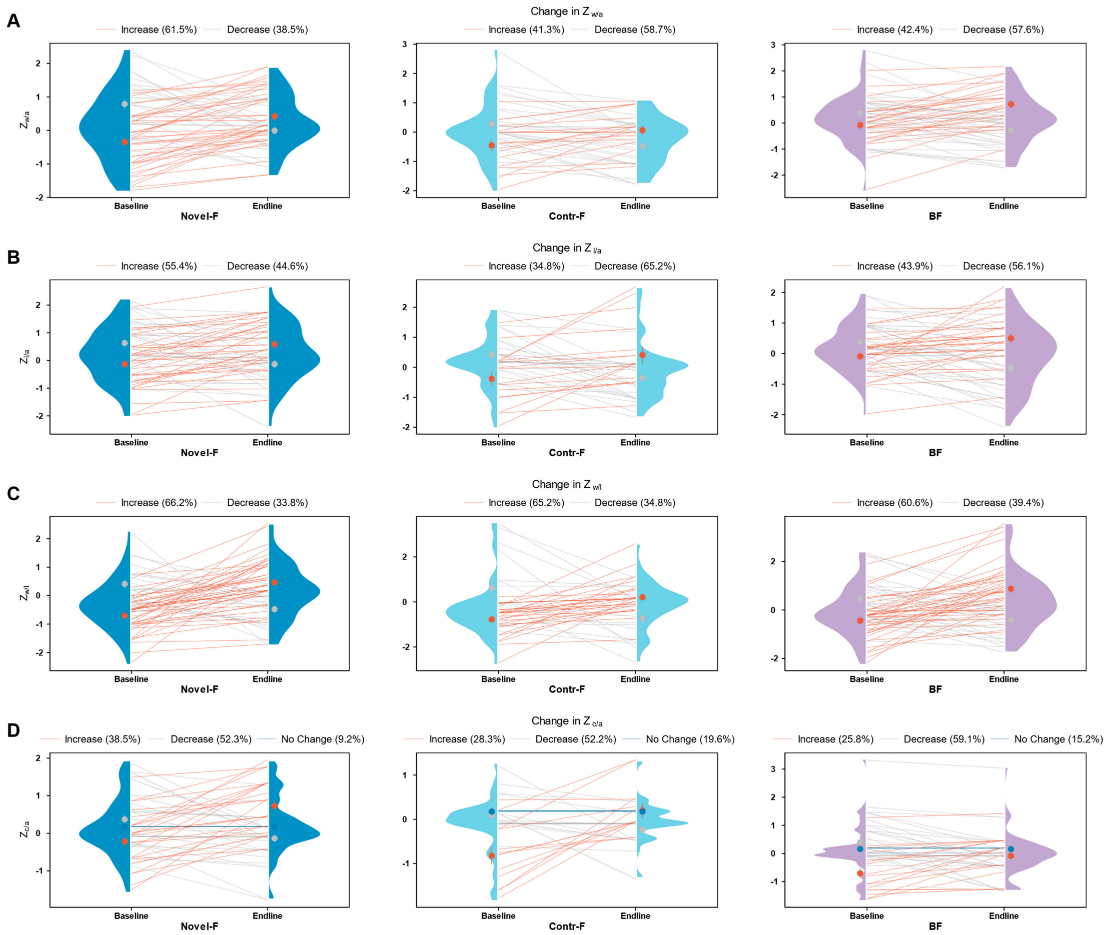

Figure S1. Z-score changes on growth measurements from baseline (30 d *postpartum*) to the end of the study (90 d *postpartum*). (A) weight-for-age z-scores ($Z_{w/a}$); (B) length-for-age z-scores ($Z_{l/a}$); (C) weight-for-length z-scores ($Z_{w/l}$); (D) head circumference-for-age z-scores ($Z_{c/a}$).

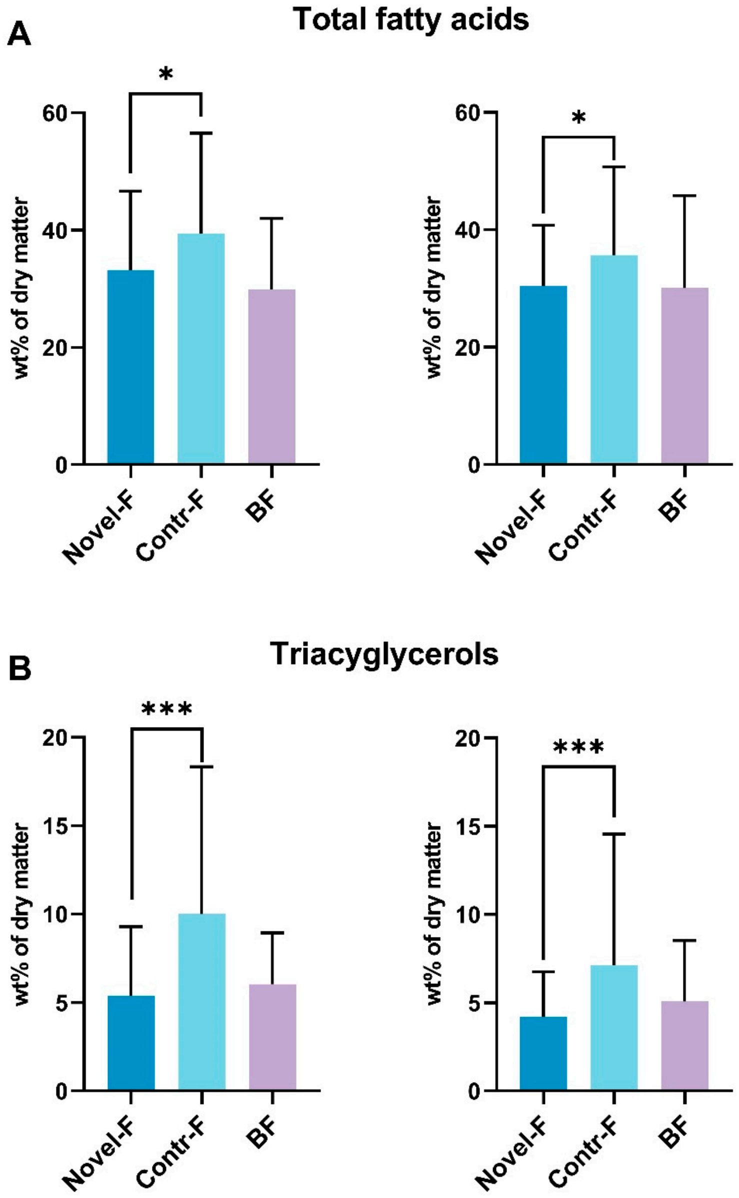

Figure S2. (A) Total fatty acids and (B) triacylglycerols (% of dry matter) infant stools measurements at 30 d *postpartum* and 90 d *postpartum*. Values were expressed as mean ± SD. One-way ANOVA was used to compare differences between groups and Bonferroni test was applied to compare the Novel-F group and the Contr-F or BF groups, respectively; *and ***indicated P<0.05 and P<0.001, respectively.
